# Agronomic performance of new open pollinated experimental lines of broccoli (*Brassica oleracea* L. var. *italica*) evaluated under organic farming

**DOI:** 10.1371/journal.pone.0196775

**Published:** 2018-05-08

**Authors:** Samira Sahamishirazi, Jens Moehring, Sabine Zikeli, Michael Fleck, Wilhelm Claupein, Simone Graeff-Hoenninger

**Affiliations:** 1 Department of Agronomy, Institute of Crop Science, University of Hohenheim, Stuttgart, Baden-Wuerttemberg, Germany; 2 Department of Biostatistics, Institute of Crop Science, University of Hohenheim, Stuttgart, Baden-Wuerttemberg, Germany; 3 Kultursaat e.V. Breeding Association, Echzell, Hesse, Germany; Agresearch Grasslands Research Centre, NEW ZEALAND

## Abstract

In order to develop new open pollinating cultivars of broccoli for organic farming, two experiments were conducted during fall 2015 and spring 2016. This study was aimed at comparing the agronomic performance of eleven new open pollinating breeding lines of broccoli to introduce new lines and to test their seasonal suitability for organic farming. Field experiments were carried out at the organic research station Kleinhohenheim of the University of Hohenheim (Stuttgart-Germany). Different agronomic traits total biomass fresh weight, head fresh weight, head diameter, hollow-stem, fresh weight harvest index and marketable yield were assessed together with commercial control cultivars. The data from both experiments were analyzed using a two-stage mixed model approach. In our study, genotype, growing season and their interaction had significant effects on most traits. Plants belonging to the fall growing season had bigger sizes in comparison to spring with significantly (p< 0.0001) higher biomass fresh weight. Some experimental lines had significant lower head fresh weight in spring in comparison to the fall season. The high temperature during the harvest period for the spring season affected the yield negatively through decreasing the firmness of broccoli heads. The low average minimum temperatures during the spring growing season lead to low biomass fresh weight but high fresh weight harvest index. Testing the seasonal suitability of all open pollinating lines showed that the considered fall season was better for broccoli production. However, the change in yield between the fall and the spring growing season was not significant for “Line 701” and “CHE-MIC”. Considering the expression of different agronomic traits, “CHE-GRE-G”, “Calinaro” and “CAN-SPB” performed the best in the fall growing season, and “CHE-GRE-G”, “CHE-GRE-A”, “CHE-BAL-A” and “CHE-MIC” and “Line 701” were best in the spring growing season, specifically due to the highest marketable yield and proportion of marketable heads.

## Introduction

According to FAO statistics [1], the production quantity of cauliflower and broccoli worldwide reached 2.4 million tons in 2014. Broccoli (*Brassica oleracea* L. var. *italica*) is an economically important vegetable. Its production and consumption has a long history in Europe, as it fits into European diets [2]. In Germany, broccoli is currently cultivated on 2170 ha, about 1100 farms are involved in its’ production and the average marketable yield is 13.6 t ha^-1^ annually [3]. Broccoli is also an important crop in organic farming (OF), albeit with lower marketable yields compared to conventional farming, with about 10 t ha^-1^ [4]. Briefly, in Germany, shares of organic vegetable production related to total vegetable production is 9%, which is in total 10.392 ha. Also, the percentage of organic vegetable consumption related to the overall vegetable consumption is approximately 6 to 7%.

Today, the broccoli cultivars that are on the market for commercial purposes are almost exclusively F1 hybrids [5]. In OF, F1 hybrids showed an average performance with regard to quality and yield [6]. It is critical in OF to develop F1 hybrids as it requires cytoplasmic male sterility (CMS) derived from Japanese radish by cell fusion as a breeding technique [7]. Some OF organizations even forbid the use of CMS-hybrids, because this practice is seen as a genetic modification that is going against the principles of organic farming [8]. Moreover, man-made hybridization in plant breeding is seen as a practice that is not in line with the principle of plant specific- and genotype integrity as it should be applied in organic breeding [9]. Hence, developing genotypes such as new open pollinating (OP) breed lines, which are considered to be heterogeneous, could be one option for organic farming [5]. Generally, F1 hybrids of broccoli produce small sized plants with big sized and uniform heads [10], which better reflect the demands of consumers and the needs of retailers. The main benefit of the production of F1 hybrids is the stability of plants across different environments [11]. These cultivars are resistant to most abiotic and biotic stressors and typically show a high degree of uniformity in color, buds, firmness and harvesting periods [12]. F1 hybrids are genetically homogeneous [13] but if farmers multiply the seeds of the F1 generation, the resulting F2 generation faces loss of hybrid vigor and is usually so heterogeneous that on-farm seed reproduction has no opportunity. Contrary to this, OP cultivars give farmers the possibility to harvest their own seeds for reproduction [5, 14]. The heterogeneous genotypes are also resistant to the influence of genetic and environmental interactions due to the heterogeneity in their genetic structures [15], also due to better genotype buffering against different growing conditions when compared to homogeneous ones [14]. Furthermore, according to Ciancaleoni et al. [10], OP genotypes show a great variability and are distinguished from each other by differing cold requirements for flower induction, sprouting habit, leaf shape, color and harvesting times. Thus, heterogeneous genotypes are particularly beneficial [14]. On the other hand, as the organic seed market is still not big enough to attract the professional plant breeding companies economically [7, 16, 17], only few cultivars have been specifically bred for OF thus far [5].

According to Renaud et al. [6], in the current market, the existing old OP cultivars of broccoli lack quality traits and uniformity. Also, considering the review study by Lammerts van Bueren et al. [7] on the necessity of breeding for organic and low-input production conditions, significant breeding efforts for the organic sector are required to support the needs of organic farmers. There have been some attempts, such as previous work of Renaud et al. [6] on breeding OP genotypes of broccoli. In order to develop new OP cultivars of broccoli for organic farming, the University of Hohenheim in cooperation with the NGO Kultursaat e.V. (organization of on-farm breeders) tested and selected commercial cultivars and new experimental lines of broccoli suitable for OF in Germany. The selection criteria were agronomic traits such as; yield level, stability of yield over time and different quality attributes associated with research focused on replacing current cultivars with new OP lines in OF. This study is specifically aimed at evaluating the agronomic performance of experimental genotype populations by comparison with commercial control cultivars in order to: (1) introduce new OP broccoli experimental populations for OF, and (2) to test the seasonal suitability of these OP genotype populations.

## Materials and methods

### Plant materials and field trials

Eleven OP breeding lines, two F1 hybrids and one OP cultivar of *Brassica oleracea* var. *italica* were tested under OF conditions during fall 2015 and spring 2016 which are listed in Table 1. The field experiments were carried out at the organic research station of the University of Hohenheim. For detailed description of field trials, please see our previous study Sahamishirazi et al. [18]. The harvest window in fall growing season was six weeks, during which, broccoli heads were harvested seven times. In spring, the harvest window was three weeks with four times of harvest (see Sahamishirazi et al. [18]). Note that for the data described above, the effects caused by the two different experiments, the two different years and the two different growing seasons are totally confounded. To simplify the further description, this confounded effect is called as growing season effect from now on.

**Table 1:** Comparison of biomass fresh weight (g), head fresh weight (g), Diameter (cm), proportion of hollow stem (%), Fresh weight harvest index (FWHI %), marketable yield (t ha^-1^) and share of marketable heads of broccoli samples within fall 2015 and spring 2016.

	Genotype	Season	Biomass fresh weight (g)	Head fresh weight* (g)	Diameter (cm)	Proportion of hollow stem^1^ %	FWHI (%)	Marketable yield (t ha^-1^)	Marketable heads (%)
**Commercial control cultivars**	Batavia F1	fall	1434.92 ± 52.46^a^	358.67 ± 11.97	11.84 ± 0.23	18.07	25.53^b^	15.32 ± 1.3^a^	87.86
		spring	804.61 ± 48.77^b^	274.61 ± 15.28	15.5 ± 1.29	0.6	35.51^a^	7.79 ± 0.6^b^	68.33
	Marathon F1	fall	1700.85 ± 61.66^a^	317.68 ± 13.74	11.94 ± 0.29	11.36	18.07^b^	12.82 ± 1.3^a^	79.21
		spring	797.77 ± 61.67^b^	260.49 ± 18.88	13.16 ± 1.6	0	32.82^a^	8.46 ± 0.6^b^	84.37
	Miranda	fall	1277.82 ± 58.74	275.67 ± 13.42	11.79 ± 0.26	28	22	10.14 ± 1.3	79.17
		spring	n.a. ^2^	No heads	No heads	No heads	n.a.	No heads	No heads
**Experimental genotype population lines**	CHE-BAL-A	fall	1356.95 ± 55.03^a^	312.51 ± 11.97	12.01 ± 0.24	13.06	23^b^	10.51 ± 1.3^a^	68.19
		spring	966.12 ± 51.59^b^	287.55 ± 16.24	12.79 ±1.37	6.62	31.04^a^	6.55 ± 0.6^b^	61.25
	CAN-SPB	fall	1124.59 ± 55.57^a^	273.63 ± 12.22	12.38 ± 0.25	25.5	24.89^b^	15.83 ± 1.3^a^	76.12
		spring	693.03 ± 56.46^b^	245.45 ± 17.65	13.00 ± 1.48	0	35.39^a^	5.38 ± 0.6^b^	60.41
	Calinaro	fall	971.94 ± 54.5^a^	274.63 ± 11.86	11.92 ± 0.24	0	28^b^	11.72 ± 1.3^a^	73.64
		spring	648.53 ± 50.14^b^	247 ± 15.76	13.13 ± 1.33	0	39.03^a^	3.75 ± 0.6^b^	42.5
	TH-COA	fall	1431.52 ± 59.63^a^	272.87 ± 12.8	12.31 ± 0.26	35.83	19.02^b^	6.72 ± 1.3^a^	62.23
		spring	915.41 ± 53.98^b^	229.11 ± 16.95	12.1 ± 1.42	2.33	25.48^a^	2.06 ± 0.6^b^	31.25
	CHE-GRE-A	fall	1141.4 ± 52.34^a^	250.22 ± 11.38	12.38 ± 0.23	22.76	23.41^b^	10.04 ± 1.3^a^	67.3
		spring	687.16 ± 48.53^b^	204.59 ± 15.26	12.64 ± 1.29	0.6	30.74^a^	6.57 ± 0.75^b^	67.91
	CHE-GRE-G	fall	1260.88 ± 57.37^a^	305.5 ± 12.51	12.32 ± 0.25	14.06	24.72^b^	15.56 ± 1.3^a^	81.54
		spring	786.67 ± 48.72^b^	253.98 ± 15.25	12.15 ± 1.28	0.6	33.15^a^	6.23 ± 0.52^b^	70.83
	TH-LIM-19-28	fall	1128.89 ± 56.46^a^	276.41 ± 12.26	11.86 ± 0.25	0	25.29^b^	7.16 ± 1.3^a^	65.68
		spring	591.75 ± 52.2^b^	223.04 ± 16.31	12.16 ± 1.37	0	38.7^a^	3.2 ± 0.6^b^	45
	TH-LIM-20-68	fall	952.97 ± 52.86^a^	255.02 ± 11.53	11.64 ± 0.22	30.63	27.31^b^	9.5 ± 1.3^a^	66.34
		spring	568.18 ± 50.15^b^	210.57 ± 15.75	13.09 ± 1.33	0.6	37.13^a^	2.06 ± 0.6^b^	32.08
	Line 124	fall	928.05 ± 49.66^a^	253.3 ± 10.78	11.33 ± 0.22	18.4	28.13^b^	8.55 ± 1.3^a^	66.83
		spring	675.38 ± 50.17^b^	242.33 ± 15.74	12.44 ± 1.33	0.6	36.94^a^	3.42 ± 0.6^b^	40.41
	Line 701	fall	1395.4 ± 67.7^a^	328.46 ± 14.75	11.68 ± 0.30	13.03	23.84^b^	4.04 ± 1.3^a^	38
		spring	676.16 ± 56.88^b^	257.29 ± 17.79	11.69 ± 1.49	0	38.79^a^	6.34 ± 0.6^a^	75.41
	CHE-MIC	fall	1212.84 ± 57.04^a^	294.95 ± 12.69	12.23 ± 0.25	34.43	24.75^b^	9.44 ± 1.3^a^	70.32
		spring	866.37 ± 52.77^b^	248.38 ± 16.4	12.83 ± 1.38	0.34	30.17^a^	6.54 ± 0.6^a^	72.91

Means of one genotype in one column followed by different letters (^a^ and ^b^) significantly different from each other (p < 0.05).

^1^ Incidence of hollow stem is rated as 0 = No, 1 = Yes, the probability of incidence of hollow stem is analyzed.

^2^ Not available

* No letter display was created simple means for this variable, as the marginal means of genotypes across growing seasons should be compared.

### Agronomic traits

#### Total biomass fresh weight, head fresh weight and head diameter

Total biomass fresh weight was recorded as the total aboveground biomass. This included the weight of stem, leaves, lateral branches and head. After weighing the total biomass, the flower head, formed in the center of the plant, was cut to 18 cm length (including stem) and the head fresh weight was measured. Harvesting was carried out once the head reached a marketable head diameter ≥ 10 cm. The diameter was recorded as the mean of a triplicate measurement of the widest part of the head using a Vernier caliper.

#### Proportion of hollow-stem

After cutting the heads to 18 cm length the existence of hollow stems was assessed. Presence or absence of a hole in the stem was scored as “positive” and “negative”.

#### Fresh weight harvest index (FWHI) and marketable yield

Fresh weight harvest index (FWHI) was defined, according to Tan et al. [19], as:

Equation 1:

$$FWHI\;=\;\frac{100\times HFW}{\left(HFW+\;weight\;of\;residual\right)}$$

Where, “HFW” is the head fresh weight and the “weight of residual” is the fresh weight of biomass excluding head weight.

In order to calculate the marketable yield, all marketable broccoli heads, which had no quality defects (such as loos buds, brownish color and etc.) and had a minimum diameter ≥10 cm were taken into consideration. Broccoli marketable yield was calculated for each population in tons per hectare as follows:

Equation 2:

$$Marketable\;yield\;=\;\frac{total\;weight\;of\;marketable\;broccoli\;heads\;(t)}{area\;(ha)}$$

To assess the performance of each line for production of marketable broccoli heads, the proportion of broccoli plants with marketable heads in relation to the total number of broccoli plants evaluated per genotype population was calculated.

### Statistical analysis

The experimental design of fall 2015 experiment was a randomized complete block design with three replicates each consisting of 14 plots. For the spring experiment 2016, plots were arranged in a resolvable row-column design [20] with 14 rows and three columns (within a column all 14 genotypes were tested, thus it corresponds to a replicate). Note that the effects of different experiments, different years and different growing seasons are totally confounded. Hence, we described and modelled the confounded effect by the growing season but still meant the confounded effect. The data for both the fall and spring experiments were analyzed using a two-stage mixed model approach [21, 22]. The stage one analysis focused on individual experiments. The stage two analysis was across the two experiments, fall and spring. Analysis of the data from the experiment in fall 2015 of the traits; total biomass fresh weight (g), head fresh weight (g), head diameter (cm) and total yield, was conducted using the mixed linear model

$$y_{ijk}\;=\;\mu +b_{k}+g_{i}+h_{j}+{\left(gh\right)}_{ij}+e_{ijk},$$ (1)

where *g*_*i*_, *h*_*j*_ and (*gh*)_*ij*_ are the fixed main effects of the *i*^th^ genotype and *j*^th^ harvest time as well as the fixed interaction effects between the *i*^th^ genotype at the *j*^th^ harvest time, respectively. *b*_*k*_ is the *k*^th^ random block effect and *e*_*ijk*_ is the error of observation *y*_*ijk*_ assuming that error effects from observations from the same plot but different harvest times are potentially correlated with a first-order autoregressive variance-covariance structure. Data from experiment in spring 2016 was analyzed using a similar model, just replacing block effects by row and column effects:

$$y_{ijkl}\;=\;\mu +{row}_{k}+{col}_{l}+g_{i}+h_{j}+{\left(gh\right)}_{ij}+e_{ijkl},$$ (2)

in which all effects are defined similar to (1). *row*_*k*_ and *col*_*l*_ are random effects for the *k*^th^ row and *l*^th^ column, respectively. For analysis of total yield, both models (1) and (2) were simplified by dropping all effects including harvest time. Note that total yield is the sum of all yields harvested on the same plot, thus no harvest effect can be estimated. For both experiments genotype-by-harvest time least square means of the *n*^th^ growing season (${\hat{\mu }}_{ijn}$) were estimated and subject to an across-growing season analysis with the following second stage model:

$${\hat{\mu }}_{ijn}\;=\;\mu +g_{i}+a_{n}+h_{ln}+{\left(ga\right)}_{in}+{\left(gh\right)}_{jln}+f_{ijln},$$ (3)

where *μ* is the general intercept, *g*_*i*_, *a*_*n*_, and (*ga*)_*in*_ are the fixed main effects of the *i*^th^ genotype, *n*^th^ growing season, *j*^th^ harvest time within growing season *n* and the interaction effects between the *i*^th^ genotype at the *n*^th^ growing season, respectively. *h*_*jn*_ and (*gh*)_*jln*_ are assumed as random effect of the *j*^th^ harvest time within growing season *n* and the interaction effects between the *i*^th^ genotype at the *j*^th^ harvest time within growing season *n*, respectively. Due to limited degrees of freedom [23], the former was formally taken as fixed in the analysis. *f*_*ijn*_ is the approximated error effect estimated in (1) or (2) for genotype-by-harvest time mean ${\hat{\mu }}_{ijn}$. To use error effects from the first stages, Smith weights [24] were calculated using a SAS macro [22]. We estimate both genotype main effects and genotype-by-growing season means from Eq (3). Data measured as a percentage was logit transformed prior to analysis. Residuals were checked graphically for normality of distribution, homogeneity of variance and potential outliers. If they latter were non-plausible, they were excluded from data previous to statistical analysis. No means of across growing seasons for cultivar “Miranda” was calculated as it did not produce any broccoli heads in spring 2016. After finding significant differences via F-test, a multiple t-test with α = 0.05 was used to compare genotype means within or across growing seasons. Note that this testing approach is called ANOVA or F test protected post hoc testing meaning that the F test ensures the family-wise error rate of 5% while t tests only ensure the comparison-wise error rate. To visualize which genotypes perform best for which trait and to show the correlations between traits, principal component analysis was performed using variety means across growing seasons of total yield and the other traits. From this analysis the first two dimensions were plotted as a biplot [25, 26, 27]. All statistical analysis of both experiments were determined by using SAS version 9.4. Additionally, graphics were generated using SigmaPlot 12.0.

## Results and discussion

### Growing and climate condition

**Fig 1:**
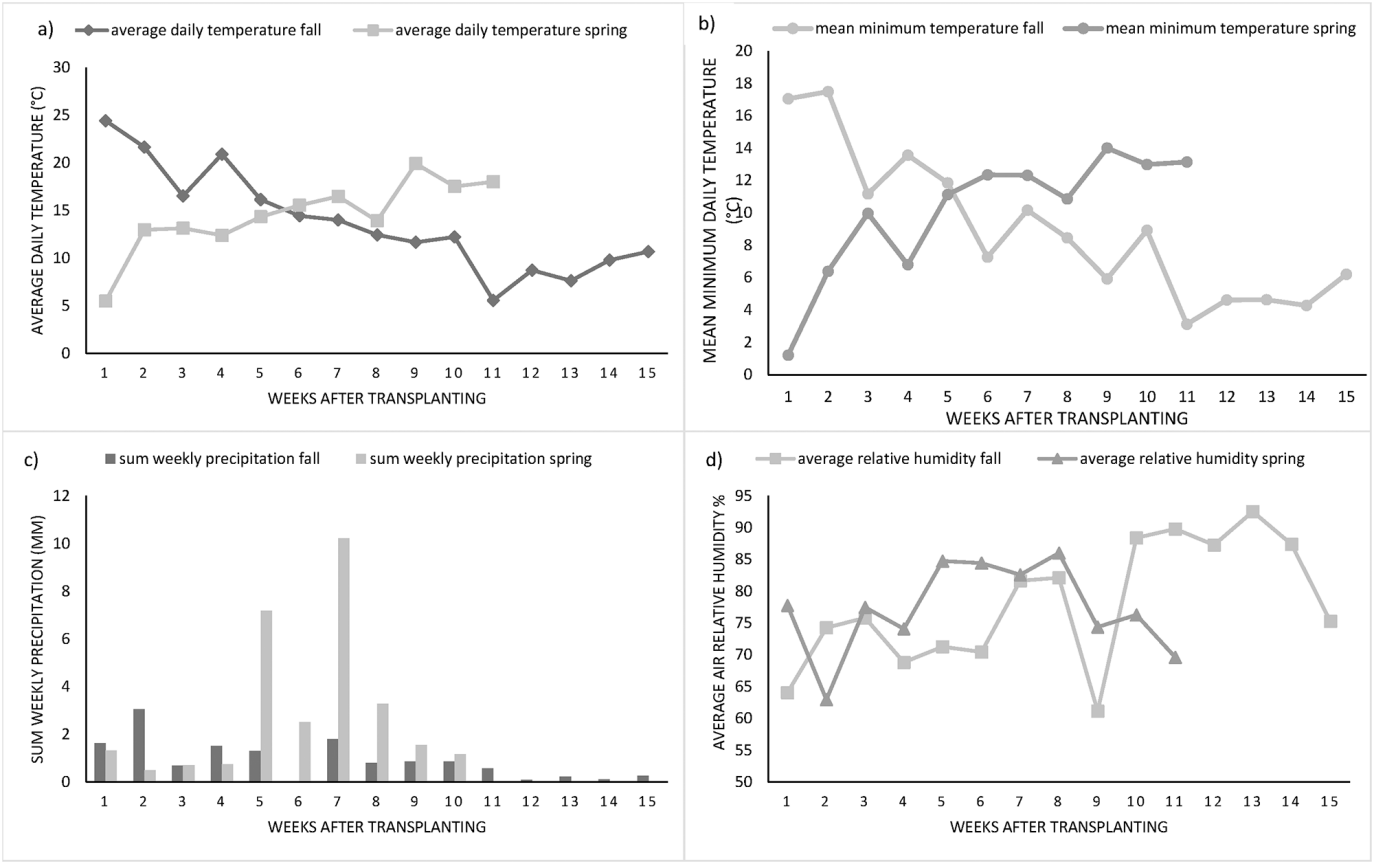
The average daily temperature (a), mean minimum daily temperature (b), sum weekly precipitation (c) and average air relative humidity (d) in the region of Hohenheim during fall 2015 and spring 2016 from transplanting to the end of harvest.

In general, cultivation period of fall and spring season lasted 15 and 11 weeks after transplanting. The 15 weeks of growth in fall 2015 included ten weeks of vegetative and generative growth and five weeks of harvesting. In spring 2016, the 11 weeks of growth contained seven weeks of vegetative and generative growth and four weeks of harvesting. The shorter cultivation time during the spring season was due to higher temperatures from the beginning of head formation to the end of harvest [28], which potentially accelerated plant development in whole. In the fall growing season 2015, the average daily temperature decreased from 22 °C, at the transplanting time in August, to 7 °C at the end of harvest in November (Fig 1a). Throughout the spring season 2016, the average daily temperature increased from 9 °C to 20 °C during April to July (from transplanting to the end of harvest). The average daily air temperature values were higher in fall season 2015 than in spring season 2016 during the stages of growth and head formation up to the beginning of harvesting. The sum of precipitation was recorded throughout both seasons (Fig 1c). According to the Fig 1c, precipitation was much higher in spring 2016 in comparison to fall 2015 during the whole growing season. Specifically, the highest precipitation was in the fifth and the seventh week after transplanting. Regarding the average relative humidity, the range was similar for both seasons from 60% to 90%, although the changing trend of the relative humidity during both seasons was much different (Fig 1d) based on the amount of precipitation.

Of the three commercial control cultivars planted in spring 2016, “Miranda” (OP) failed to produce any heads. Similar to the study reported by Farnham et al. [29], some broccoli cultivars did not form heads due to the high temperatures. We assume that “Miranda” showed a similar response and therefore was sensitive to high temperatures during the spring trial. Even though the central stem was formed, no head was produced at all. Nevertheless, in fall 2015, “Miranda” performed very well with a mean biomass weight of 1278 g and mean head weight of 275.7 g (Table 1).

### Total biomass fresh weight

Generally, plants in the fall growing season 2015 were bigger in size in comparison to plants in spring 2016. According to Table 1, total biomass fresh weight per broccoli plant in fall 2015 ranged from 928 g (Line 124) to 1700 g (Marathon F1). This range in spring 2016 was significantly lower than in the fall season 2015, between 568 g (TH-LIM-20-68) and 966 g (CHE-BAL-A). Across the two growing seasons the “CHE-BAL-A” had significantly higher biomass weight than the commercial cultivars, as well as the other lines except for “TH-COA” and “CHE-MIC” (Table 1). According to the study by Tan et al. [19], the decrease of the average minimum temperatures during the growing season led to a decrease of biomass fresh weight. Likewise, in the current study, the mean minimum temperatures of the growing season in fall 2015 were higher than in spring 2016 during the first five weeks after transplanting (Fig 1b). The lower minimum temperatures in spring (-4 °C to 3.3 °C) resulted in significantly lower biomass weight in comparison to fall (6.6 °C to 8.1 °C) for all genotype populations and also lead to shorter cultivation periods (Table 1). Results from ANOVA, in Table 2, showed significant effects (p<0.0001) of genotype × growing season interaction and growing season × harvest interaction on biomass fresh weight.

**Table 2:** Results from the analysis of variance for different agronomic traits.

Effects	Biomass fresh weight	Head weight	Diameter	Hollow stem	FWHI^1^	Marketable yield
**Genotype**	<0.0001	<0.0001	n.s. ^2^	n.s.	<0.0001	<0.0001
**Growing season**	<0.0001	<0.0001	n.s.	n.s.	<0.0001	<0.0001
**Genotype × Growing season**	<0.0001	n.s.	n.s.	n.s.	0.0232	<0.0001
**Growing season × harvest**	<0.0001	0.5086	<0.0001	n.s.	0.0478	-

^1^ Fresh Weight Harvest Index

^2^ Not significance

### Head diameter

Diameter measurements of marketable heads indicated a range from 11.33 cm (Line 124) to 12.38 cm (CAN-SPB) and from 11.69 cm (Line 701) to 15.5 cm (Batavia F1) in fall 2015 and spring 2016, respectively (Table 1). However, according to Table 2, for this trait no significant effects for genotype, growing season and their interactions were found (p > 0.05). Yet, a significant effect of harvest time within growing seasons on head diameter was observed (p < 0.0001), which was caused by higher diameter of broccoli heads harvested later in the growing season.

### Proportion of hollow stems

According to Table 1, the proportion of hollow stem in fall 2015 ranged from 0% (Calinaro, TH-LIM-19-28) to 36% (TH-COA). The range in spring 2016 was from 0% (Calinaro, CAN-SPB, TH-LIM-19-28, Line 701 and Marathon) to 7% (CHE-BAL-A). Similar to the head diameter, and according to the results of ANOVA (Table 2), this trait was not significantly influenced by genotype, growing season and their interactions (p > 0.05). The comparison of the proportion of hollow stem for each genotype across the two consecutive growing seasons showed a non-significant decrease of the proportion of hollow stems in spring compared to fall. Occurrence of hollow stem as a physiological disorder is not desirable in broccoli as it has negative effects on the shelf life. Environmental factors like rapid growth rate [30, 31], high nitrogen fertilization [32, 33] and lower plant density [34] increase the incidence of hollow stem. While the level of nitrogen fertilization was set to 300 kg ha^-1^ in both cropping periods, plant density was lower in spring season. Conversely, in the current research, the proportion of hollow stem was less in spring in comparison with fall growing season.

### Fresh weight harvest index (FWHI) and marketable yield

According to Table 1, the FWHI ranged from 18% (Marathon F1) to 28% (Calinaro) in fall 2015. The range in spring 2016 was between 26% (TH-COA) and 39% (Calinaro) which was higher than fall. Outcomes of ANOVA (Table 2) showed significant effect of genotype × growing season interaction and harvest date on FWHI trait (p = 0.0232 and p = 0.0478, respectively). According to Tan et al. [19], low average minimum temperatures during the growing season lead to low biomass fresh weight but high FWHI. Also, Kaluzewicz et al. [28] stated that FWHI increased with later planting time. In the current study, the mean minimum temperatures during fall 2015 were higher than in spring 2016 during the first five weeks after transplanting (Fig 1b). Therefore, the lower air temperatures in spring could result in lower biomass weight and significantly higher FWHI in comparison to fall. We have to consider that in 2016 only spring cropping was applied, therefore the air and soil temperature was lower during day and night. Although soil temperatures have not been monitored in our study, we are sure that soils are colder in April than in July. The warmer the soil (with comparable water and air conditions) the more the soil will be mineralized. In case of the OP breeding lines “CHE-BAL-A”, “CAN-SPB” and “Calinaro”, significantly lower biomass weight resulted in significantly higher FWHI as the head weight was not significantly different across fall and spring. The genotype populations with higher FWHI values are useful for commercial production as they produced heavy broccoli heads in combination with low residual weight. Hence, the OP breeding lines could be good choices for cropping as they reached the highest FWHI values in both fall 2015 and spring 2016.

Results of evaluation of marketable yield of each genotype population are shown in Table 1. In growing season fall 2015, the marketable yield ranged from 4.0 t ha^-1^ (Line 701) to 15.8 t ha^-1^ (CAN-SPB). The range of marketable yield decreased in growing season spring 2016 and varied from 2.1 t ha^-1^ (TH-COA) to 8.5 t ha^-1^ (Marathon F1). All genotypes had significantly higher yield in fall 2015 compared to spring 2016. However, the yield reduction between the fall and the spring growing seasons was not significant for “Line 701” and “CHE-MIC” (Table 1). Statistical analysis (Table 2) showed significance of genotype × growing season interactions for marketable yield of broccoli heads (p < 0.0001). Similar to results reported by Pek et al. [35], significantly higher yields were achieved in our study during the fall growing season except for “Line 701”. Our results were in line with the outcomes of Elwan and Abd-Elhamed [36] which showed higher broccoli yields in the fall compared to spring. Likewise, Tan et al. [37] observed lower yields in spring compared to fall in previous studies. The significant effect of growing season may be caused by the high dependency of broccoli yield on temperatures [28]. Higher temperature in spring compared to fall results in a decrease of photosynthetic rate and increase in respiratory losses which may lead to yield losses [38]. Higher yields will be obtained when the temperature ranges between 15 to 25 °C during an early stage after cultivation and during the phase which proceeds to harvest [28, 39]. According to a study by Kaluzewicz et al. [28], the longer the broccoli plants are exposed to the temperatures of 15–25 °C, the higher the yield. The same authors found that temperatures between 25 to 30 °C during the harvest period results in lower yield. More precisely, according to previous studies and conforming to practical experiences, high temperature during the harvesting period affected the firmness of broccoli heads negatively, specifically formation of loose broccoli heads increased when the temperature rose above 18 °C [28, 40]. Similarly, we observed the negative effect of higher temperature, which resulted in loose broccoli heads in the samples of the spring growing season, hence obtained lower yield.

### Head fresh weight

Generally, the experimental lines had significantly lower head fresh weight in spring in comparison to the fall season (Table 1). Tan et al. [19] reported that the overall quality of broccoli heads was mostly influenced by genotype but only slightly by the environment [29]. In this regard, we observed significant effects of genotypes on the head fresh weight in our study (Table 2). The effect of growing season was significant on this trait as well. According to Table 2, since the interaction of genotype and growing season did not affect the head weight significantly, the values across growing seasons is provided for this trait in Table 3. Comparison of the mean head fresh weight of genotypes across fall and spring growing seasons showed that the OP lines “CHE-BAL-A” and “Line 701” had significantly heavier heads than the other lines except for “CHE-GRE-G” and “CHE-MIC”. The performance of these two lines regarding head weight trait were similar to the commercial control cultivars.

**Table 3:** Comparison of the mean values of head fresh weight (g) of different broccoli samples across growing seasons (fall 2015 and spring 2016).

	Genotypes	Head fresh weight (g)
**Commercial control cultivars**	Batavia F1	316.64^a^
	Marathon F1	289.08^abc^
	Miranda	n.a.^1^
**Experimental genotype population lines**	CHE-BAL-A	300.03^ab^
	CAN-SPB	259.54^defg^
	Calinaro	260.82^cdef^
	TH-COA	250.99^defg^
	CHE-GRE-A	227.41^g^
	CHE-GRE-G	279.74^bcd^
	TH-LIM-19-28	249.73^efg^
	TH-LIM-20-68	232.79^fg^
	Line 124	247.81^efg^
	Line 701	292.88^ab^
	CHE-MIC	271.66^bcde^

Means in one column followed by different letters significantly different from each other (p < 0.05).

^1^ Not available

**Fig 2:**
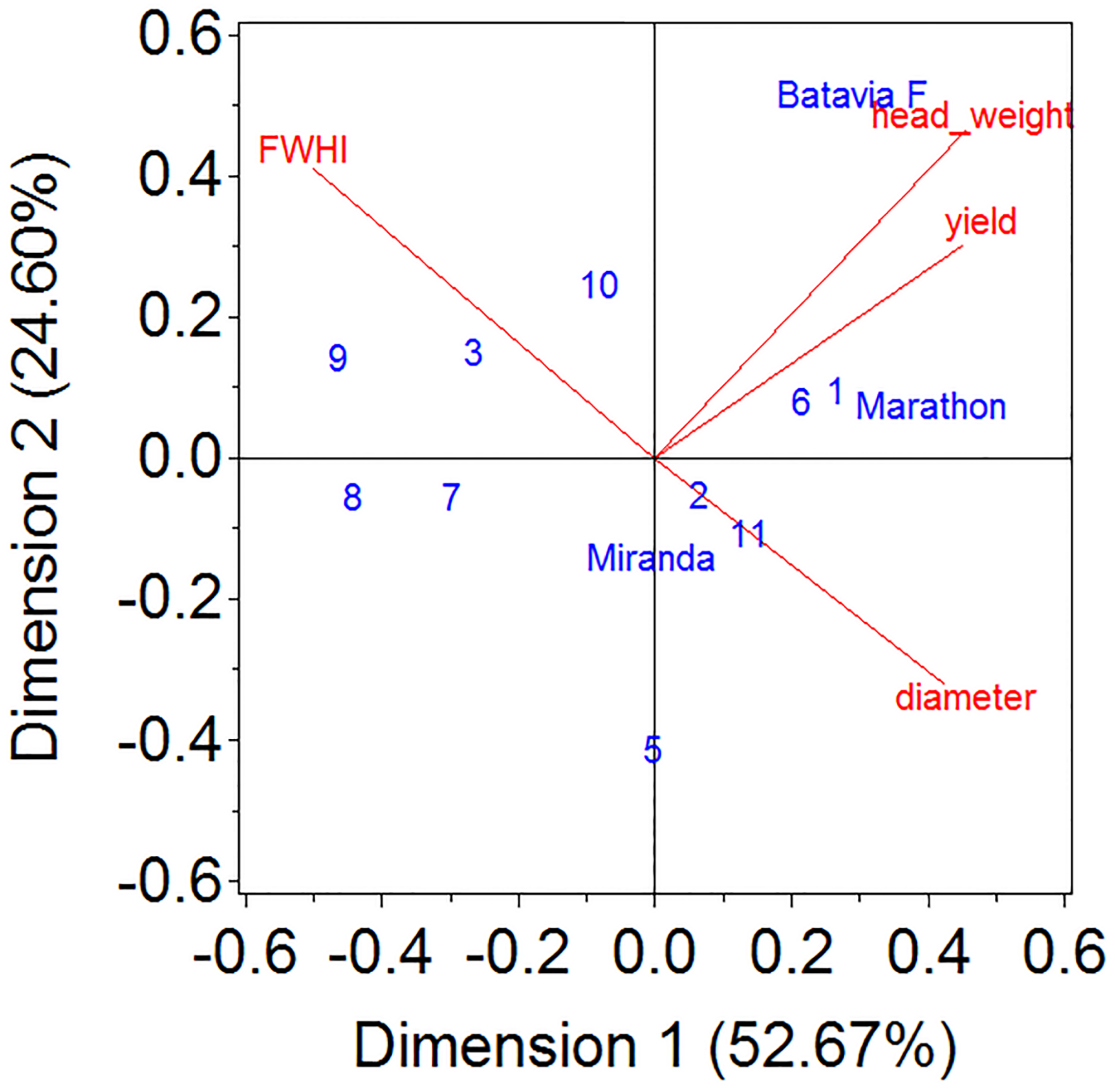
Biplot of genotype-by-trait means across growing seasons. Arrows denote traits, lines names denote experimental lines. 1: CHE-BAL-A, 2: CAN-SPB, 3: Calinaro, 4: TH-COA, 5: CHE-GRE-A, 6: CHE-GRE-G, 7: TH-LIM-19-28, 8: TH-LIM-20-68, 9: Line 124, 10: Line 701, 11: CHE-MIC.

This research assessed the agronomic performance of OP breeding lines compared to control cultivars (hybrids and released OP) during fall 2015 and spring 2016 growing seasons as well as across both growing seasons. For the latter, correlations between traits and genotype-by-trait interactions can be seen in the biplot (Fig 2). The biplot represents 77.27% of the total variance and therefore just approximate correlations between traits or genotype-by-trait interactions. The FWHI and diameter are negatively correlated, yield and head weight are positively correlated. Biomass weight and yield showed high positive correlations as their red arrows pointing in the same direction. Batavia had positive interaction effects with yield as its projection on trait arrows is positive (in the direction of the arrow). In general, the plot shows that correlations between traits are most often low and vary from negative to positive correlations.

Overall, the environmental conditions in growing season fall 2015 resulted in significantly higher yields, head and biomass fresh weight compared to the growing season spring 2016. The results showed that despite a large variability within the newly bred OP lines, some of the OPs already performed similar to the hybrid cultivars, frequently used in organic farming, regarding different agronomic traits such as head fresh weight, head diameter and etc. In the fall growing season, all of the OP lines showed 23% to 73% lower yields compared to the hybrid cultivars except for “CHE-GRE-G” and “CAN-SPB” which had non-significant different yield as hybrids. In the growing season spring 2016, all the OP lines showed 16% to 73% lower yield in comparison with hybrids. Considering the yield of the different broccoli lines, testing the seasonal suitability of all OP lines showed that the considered fall season was better suited for cultivation and production. Based on the expression of the different agronomic traits measured, OP lines “Line 701”, “CHE-BAL-A”, “CHE-GRE-G”, “Calinaro” and “CAN-SPB” performed best for cultivation in the fall growing season. However, focusing on yield performance of the experimental lines only, we would like to emphasize on “CHE-GRE-G”, “CAN-SPB” and “Calinaro” for cultivation in fall growing season. These lines had the highest ranking for marketable yield and proportion of marketable heads. Additionally, they had the shortest duration of harvest. Specifically, “CHE-GRE-G”, “CAN-SPB” performed the best in growing season fall and the yields of these two experimental lines were even higher than those of the control hybrid and released OP cultivars. In addition, suitable lines for the spring growing season based on general agronomic performance could be “Calinaro”, “CHE-MIC”, “Line 701” and “CHE-GRE-G”. Experimental lines “CHE-GRE-A”, “CHE-BAL-A” and “CHE-MIC” and “Line 701” show highest marketable yield and portion of marketable heads in the spring growing season. However, these lines still lack the requested head firmness. Therefore, this trait should be taken into account in further breeding.

Out of the experimental lines, “CHE-GRE-G” and “Calinaro” have been released and are being cultivated by local farmers and home gardeners. We would like to encourage the breeders that further genetic improvement of the proposed experimental lines would result in final broccoli cultivars, which are specifically bred for organic farming. However, when selecting lines for future breeding, other traits in addition to agronomic performance, such as health associated compounds (see Sahamishirazi et al. [18]) and sensory quality should be considered.
